# The Changes in Blood Flow Seen in the Eye after Foot Acupuncture Treatment in Mice

**DOI:** 10.1155/2020/6405471

**Published:** 2020-04-06

**Authors:** Anri Nishinaka, Koki Nitta, Takashi Seki, Hideaki Hara, Masamitsu Shimazawa

**Affiliations:** ^1^Molecular Pharmacology, Department of Biofunctional Evaluation, Gifu Pharmaceutical University, Gifu, Japan; ^2^Department of Internal Medicine & Rehabilitation Science, Tohoku University Graduate School of Medicine, Sendai, Miyagi, Japan

## Abstract

Acupuncture is used to treat a wide variety of eye diseases, although there is little evidence about the effects of acupuncture treatment and the mechanisms responsible for them. Foot acupuncture treatment has effects in both mice and humans. The purpose of this study was to investigate the effects of acupuncture treatment on ocular blood flow in mice. We evaluated ocular blood flow in C57BL/6J mice after foot acupuncture treatment using laser speckle flowgraphy. The mean blur rate, which is an index of blood flow velocity, was increased in the foot acupuncture group. Our results showed that, after 3 minutes' foot acupuncture, ocular blood flow was significantly increased in both the blood vessels and tissue of the eye in C57BL/6J mice. Thus, performing acupuncture in mice might help to determine its effects. Furthermore, acupuncture is considered to be a possible treatment for ocular disease.

## 1. Introduction

Acupuncture is a traditional Chinese medicine, which has been used for over 5,000 years [1]. It has a variety of effects on degenerative and psychosomatic diseases involving cerebral or peripheral ischemia [2–6]. In previous studies, acupuncture stimulation increased the blood flow in several organs by modulating the central circulatory system and autonomic nervous system dysfunction [7, 8]. In fact, some clinical studies have reported that intraocular pressure was reduced in glaucoma patients and visual acuity was improved in nystagmus patients after acupuncture treatment [9, 10]. In other studies, electrostimulation improved the vestibulo-ocular reflexes of rabbits with vertebrobasilar insufficiency, by inducing improvements in basilar artery hemodynamics, inner ear blood flow, and blood viscosity [7]. However, it is still poorly understood as a potential therapy for ocular disease because there is a lack of reliable evidence about its effects. In clinical studies, it is difficult to verify the effects of acupuncture treatment on ocular disease in detail due to the complexity of patients' conditions. Experimental animals can be useful for studying the effects, potential toxicities, and mechanisms of new treatments [11, 12]. It has been reported that acupuncture targeting the acupuncture point has the same effects in mice and humans [13]. Therefore, the effects of acupuncture and electrostimulation can be examined using mice.

The aim of this study was to investigate the changes in blood vessel and tissue blood flow seen in the eyes of mice after foot acupuncture treatment.

## 2. Materials and Methods

### 2.1. Study Approval

All experimental procedures were performed in accordance with the Association for Research in Vision and Ophthalmology Statement (ARVO) statement for the Use of Animals in Ophthalmic and Vision Research, and the experimental protocols were approved and monitored by the institutional animal care and use committee of Gifu Pharmaceutical University.

### 2.2. Animals

Eight-week-old male C57BL/6J mice were purchased from Japan SLC (Hamamatsu, Japan). The C57BL/6J mice are the all most widely used inbred strain in biomedical research. All animals were housed in a controlled environment (at 24°C ± 2°C under a 12-hour light/12-hour dark cycle) and received standard laboratory food and filtered clean water *ad libitum*. Twenty mice were divided randomly into two groups: the sham acupuncture group and the foot acupuncture group before acupuncture treatment.

### 2.3. Acupuncture Treatment

**Figure 1:**
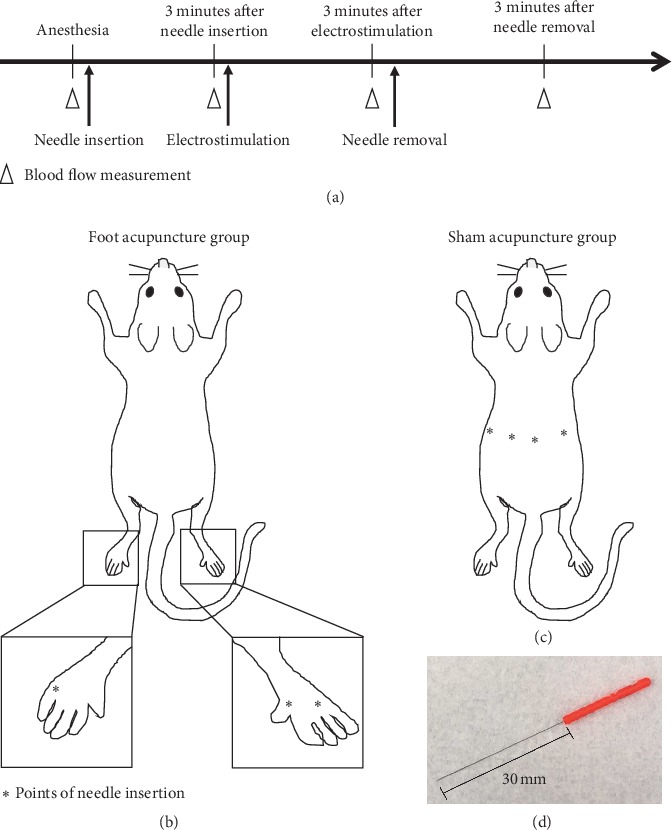
(a) Ocular blood flow was measured by laser speckle flowgraphy under anesthesia. The acupuncture was performed by inserting disposable stainless-steel needles to a depth of approximately 3 mm for 3 minutes. After 3 minutes, the mice were electrically stimulated at 7 mA for 3 minutes using the Picorina, which is an electrical stimulation device that is specifically designed for acupuncture treatment. The needles were removed after the electrical stimulation. Ocular blood flow was measured after each treatment. (b) In the foot acupuncture group, needles were inserted between the thumb and pointer finger and between the annular and little fingers on both feet, which this acupuncture points were used as the treatment for ocular disease. (c) In the sham group, acupuncture needles were inserted into the backs of mice.

The mice were anesthetized with a mixture of ketamine (80 mg/kg; Daiichi-Sankyo, Tokyo, Japan) and xylazine (6 mg/kg; Bayer HealthCare, Osaka, Japan). The acupuncture was performed by inserting disposable stainless-steel needles (0.16 mm × 30 mm; Seirin Co. Ltd., Shizuoka, Japan) to a depth of approximately 3 mm. The Picorina (Seirin Co. Ltd., Shizuoka, Japan) is an electrical stimulation device, which was specifically designed for use during acupuncture treatment. The study protocol was as follows (see Figure 1(a)): Acupuncture was performed for 3 minutes under anesthesia, which was induced using ketamine and xylazine. In the foot acupuncture group, acupuncture was performed between the thumb and pointer finger and between the annular and little fingers on both feet (see Figure 1(b)). After 3 minutes, the mice were electrically stimulated at 7 mA for 3 minutes using the Picorina, which was equipped with a clip that was specifically developed for use with a needle electrode. The needle was removed after the electrical stimulation. In the sham acupuncture group, acupuncture was performed on the backs of the mice about 4 cm from the tail using the same technique as in the foot acupuncture group, and ocular blood flow was measured under the same conditions as in the foot acupuncture group (see Figure 1(c)).

### 2.4. Ocular Blood Flow Measurements Obtained Using Laser Speckle Flowgraphy

In the present study, ocular blood flow in the choroidal and retinal regions was assessed in detail using laser speckle flowgraphy (LSFG; Softcare Co., Ltd., Fukuoka, Japan) [14–17]. Ocular blood flow was analyzed before the acupuncture treatment and after the acupuncture treatment, electrical stimulation, and the removal of the needle (see Figure 1(a)). The pupils of the mice were dilated with 1% tropicamide and 2.5% phenylephrine (Mydrin-P; Santen Pharmaceuticals Co., Ltd, Osaka, Japan) under anesthesia, which was induced with ketamine and xylazine. After that, hydroxyethyl cellulose gel (Scopisol; Senju Pharmaceutical Co. Ltd., Osaka, Japan) was applied to the cornea to prevent desiccation. The mean blur rate (MBR), which is an index of blood flow velocity, was used as the main output parameter of the LSFG [18]. A total of 118 images were used to determine the MBR. The images were continuously taken at a rate of 30 frames per second over a time period of approximately 4 seconds during one LSFG scan. After the image acquisition, the areas of blood vessels and tissue within the optic nerve head area were automatically detected using LSFG Analyzer software (version 3.1.14.0; Software Co., Ltd. Fukuoka, Japan). The MBR was determined for the choroidal and retinal regions (MA: the MBR of all areas), the blood vessels in the choroidal and retinal regions (MV: the MBR of the vasculature), and the tissue in the choroidal and retinal regions (MT: the MBR of the tissue).

### 2.5. Statistical Analyses

The data are presented as the mean ± standard error of the mean (SEM). The significance of differences was determined using the Mann–Whitney *U*-test. The statistical package for the social sciences 15.0 J for windows software (SPSS Japan Inc., Tokyo, Japan) was used for the statistical analyses. *P* values <0.05 were considered to be statistically significant.

## 3. Results

### 3.1. Increased Ocular Blood Flow Was Seen in the Mice after Foot Acupuncture Treatment

**Figure 2:**
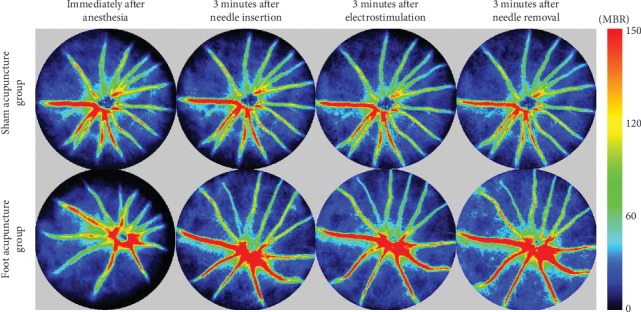
Effects of acupuncture treatment on ocular blood flow in mice. Color-coded laser speckle images of representative eyes from the foot and sham acupuncture groups are shown. The mean blur rate (MBR) is an index of blood flow velocity. The red color indicates high MBR values, and the blue color indicates low MBR values. In the foot acupuncture group, the MBR values observed after 3 minutes' acupuncture treatment were higher than those seen before the acupuncture treatment. The MBR values of the sham acupuncture group were not affected by the acupuncture treatment.

To determine the effects of foot acupuncture treatment on ocular blood flow in mice, LSFG was used to measure ocular blood flow in C57BL/6J mice. Specifically, LSFG was used to analyze blood flow in the choroidal and retinal regions based on examinations of the MBR. In Figure 2, the red color indicates high MBR values, and the blue color indicates low MBR values. In the foot acupuncture group, the MBR values observed after 3 minutes' acupuncture treatment were higher than those seen before the acupuncture treatment (see Figure 2). On the other hand, in the sham acupuncture group, ocular blood flow was not affected by the insertion of acupuncture needles into the backs of the mice (see Figure 2).

### 3.2. Increased Ocular Blood Flow Was Seen in the Blood Vessels and Tissues of the Eye in Mice after Foot Acupuncture Treatment

**Figure 3:**
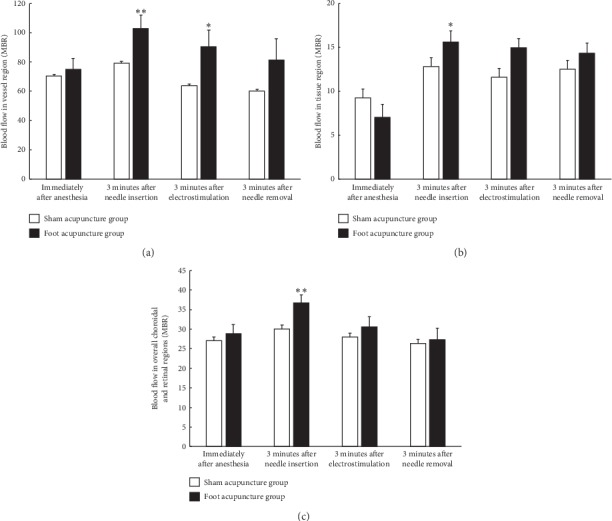
Effects of foot acupuncture on ocular blood flow in the tissue and blood vessels of the choroidal and retinal region in mice. The graph shows quantitative data obtained *via* ocular blood flow analysis of the mean blur rate (MBR) of the blood vessels in the choroidal and retinal regions (MV) (a), the MBR of the tissue in the choroidal and retinal regions (MT) (b), and the overall MBR for the choroidal and retinal regions (MA) (c). MV, MT, and MA all significantly increased after the foot acupuncture treatment. Electroacupuncture treatment did not affect ocular blood flow in the mice. Data are shown as the mean ± SEM (*n* = 10). ^*∗*^*P* < 0.05; ^*∗∗*^*P* < 0.01 versus the sham acupuncture group (Mann–Whitney *U*-test).

To determine the sites at which ocular blood flow increased in the mice after the acupuncture treatment, we manually examined the blood flow in the choroidal and retinal regions *via* LSFG analysis. The overall MBR of the choroidal and retinal regions was measured, as was the MBR of the blood vessels (MV) and tissue (MT) in the choroidal and retinal regions. Compared with that seen in the sham acupuncture group, the ocular blood flow of the foot acupuncture group was increased in both the blood vessels and tissues of the choroidal and retinal regions after 3 minutes' acupuncture treatment (see Figures 3(a) and 3(b)). Moreover, the overall MBR (MA) was significantly increased in the foot acupuncture group after 3 minutes' acupuncture treatment (see Figure 3(c)). However, no changes in ocular blood flow were seen after electrostimulation or the removal of the needles.

## 4. Discussion

This study showed the significant increase of ocular blood flow in mice after foot acupuncture. Specifically, increases in ocular blood flow were seen in both the tissue and blood vessels of the eyes in mice.

It has been reported that acupuncture targeting the vision-related acupuncture points around the orbits and on the limbs increased blood flow through the supratrochlear artery, which is a branch of the ophthalmic artery, by inducing changes in vascular resistance [19]. In glaucoma patients, foot acupuncture treatment reduced the increase in intraocular pressure seen at 15 minutes after the foot acupuncture at 1, 2, and 5 weeks by activating the autonomic nervous system [9]. Therefore, we selected the foot as the site of acupuncture treatment. In the present study, foot acupuncture treatment increased blood flow in the blood vessels and tissue of the eye in mice (Figures 2 and 3). Acupuncture increases the release of the vasodilator nitric oxide (NO) in the skin, which improves the local circulation and contributes to local warmness and the beneficial effects of acupuncture, such as pain relief and improvements in sweating and inflammation [20]. The NO produced *via* endothelial nitric oxide synthase (eNOS) is a fundamental determinant of cardiovascular homeostasis, and it helps to maintain the systemic blood pressure, vascular remodeling, and angiogenesis [21]. Therefore, the increases in blood flow seen in ocular tissue and blood vessels after foot acupuncture treatment might have been caused by vasodilation through the eNOS signaling pathway.

One acupuncture stimulation method involves the application of electrical stimulation [22, 23]. The frequency and intensity of the electrical stimulus are known to influence the effects of electrostimulation [24]. It has been reported that both low-frequency (2–4 Hz) and high-frequency (80–100 Hz) electrostimulation increase pain thresholds [24]. For example, low-frequency electrostimulation increased ovarian blood flow *via* a reflex response involving the ovarian sympathetic nerves [24]. The blood pressure in the mice was not changed after the electrical stimulation intensity in the previous studies [24]. Therefore, we selected 7 mA, a low frequency, as the electrical stimulation intensity based on the findings of previous studies. The optimal duration of electrostimulation was investigated at the same time as the effects of foot acupuncture. However, in the present study, performing electrostimulation after foot acupuncture did not increase ocular blood flow in mice. In previous studies, cerebral blood flow was improved by 20 minutes' electrostimulation in patients that had suffered acute ischemic strokes [25]. Therefore, ocular blood flow might not be altered by a short period of electrostimulation.

We consider that it is not sufficient to investigate the mechanisms for the effects of acupuncture treatment. Therefore, it is important that some animal models of ocular diseases are determined the change of the blood pressure and the mechanisms underlying these effects after foot acupuncture treatment.

We conclude that performing acupuncture treatment in C57BL/6J mice might help to determine the effects of acupuncture treatment and the mechanisms underlying these effects. In addition, acupuncture might be a useful treatment for ocular disease.

## Data Availability

The data used to support the findings of this study are included within the article.

## References

[1] Mao J. J., Kapur R. (2010). Acupuncture in primary care. *Primary Care: Clinics in Office Practice*.

[2] Blechschmidt T., Krumsiek M., Todorova M. G. (2017). Acupuncture benefits for flammer syndrome in individuals with inherited diseases of the retina. *EPMA Journal*.

[3] Blechschmidt T., Krumsiek M., Todorova M. G. (2017). The effect of acupuncture on visual function in patients with congenital and acquired nystagmus. *Medicines*.

[4] Liang X. B., Liu X. Y., Li F. Q. (2002). Long-term high-frequency electro-acupuncture stimulation prevents neuronal degeneration and up-regulates BDNF mRNA in the substantia nigra and ventral tegmental area following medial forebrain bundle axotomy. *Molecular Brain Research*.

[5] Blekher T., Yamada T., Yee R. D., Abel L. A. (1998). Effects of acupuncture on foveation characteristics in congenital nystagmus. *British Journal of Ophthalmology*.

[6] Sheth N. V., Dell’Osso L. F., Leigh R. J., Van Doren C. L., Peckman H. P. (1995). The effects of afferent stimulation on congenital nystagmus foveation periods. *Vision Research*.

[7] Watanabe M., Takayama S., Yamamoto Y., Nagase S., Seki T., Yaegashi N. (2012). Haemodynamic changes in the superior mesenteric artery induced by acupuncture stimulation on the lower limbs. *Evidence-Based Complementary and Alternative Medicine*.

[8] Sakatani K., Kitagawa T., Aoyama N., Sasaki M. (2010). Effects of acupuncture on autonomic nervous function and prefrontal cortex activity. *Advances in Experimental Medicine and Biology*.

[9] Kurusu M., Watanabe K., Nakazawa T. (2005). Acupuncture for patients with glaucoma. *Explore*.

[10] Takayama S., Seki T., Nakazawa T. (2011). Short-term effects of acupuncture on open-angle glaucoma in retrobulbar circulation: additional therapy to standard medication. *Evidence-Based Complementary and Alternative Medicine*.

[11] Fletcher E. L., Jobling A. I., Vessey K. A., Luu C., Guymer R. H., Baird P. N. (2011). Animal models of retinal disease. *Progress in Molecular Biology and Translational Science*.

[12] Khayat M., Lois N., Williams M., Stitt A. W. (2017). Animal models of retinal vein occlusion. *Investigative Opthalmology & Visual Science*.

[13] Goldman N., Chen M., Fujita T. (2010). Adenosine A1 receptors mediate local anti-nociceptive effects of acupuncture. *Nature Neuroscience*.

[14] Nishinaka A., Fuma S., Inoue Y., Shimazawa M., Hara H. (2017). Effects of kallidinogenase on retinal edema and size of non-perfused areas in mice with retinal vein occlusion. *Journal of Pharmacological Sciences*.

[15] Nishinaka A., Inoue Y., Fuma S. (2018). Pathophysiological role of VEGF on retinal edema and nonperfused areas in mouse eyes with retinal vein occlusion. *Investigative Opthalmology & Visual Science*.

[16] Fuma S., Nishinaka A., Inoue Y. (2017). A pharmacological approach in newly established retinal vein occlusion model. *Scientific Reports*.

[17] Ogishima H., Nakamura S., Nakanishi T. (2011). Ligation of the pterygopalatine and external carotid arteries induces ischemic damage in the murine retina. *Investigative Opthalmology & Visual Science*.

[18] Wang L., Cull G. A., Piper C., Burgoyne C. F., Fortune B. (2012). Anterior and posterior optic nerve head blood flow in nonhuman primate experimental glaucoma model measured by laser speckle imaging technique and microsphere method. *Investigative Opthalmology & Visual Science*.

[19] Takayama S., Seki T., Watanabe M. (2010). Brief effect of acupuncture on the peripheral arterial system of the upper limb and systemic hemodynamics in humans. *The Journal of Alternative and Complementary Medicine*.

[20] Tsuchiya M., Sato E. F., Inoue M., Asada A. (2007). Acupuncture enhances generation of nitric oxide and increases local circulation. *Anesthesia & Analgesia*.

[21] Schmetterer L. (2001). Role of nitric oxide in the control of ocular blood flow. *Progress in Retinal and Eye Research*.

[22] Uchida S., Kagitani F., Suzuki A., Aikawa Y. (2000). Effect of acupuncture-like stimulation on cortical cerebral blood flow in anesthetized rats. *The Japanese Journal of Physiology*.

[23] Onda A., Jiao Q., Nagano Y. (2011). Acupuncture ameliorated skeletal muscle atrophy induced by hindlimb suspension in mice. *Biochemical and Biophysical Research Communications*.

[24] Kim J. H., Choi K. H., Jang Y. J. (2013). Electroacupuncture acutely improves cerebral blood flow and attenuates moderate ischemic injury via an endothelial mechanism in mice. *PLoS One*.

[25] Stener-Victorin E., Kobayashi R., Kurosawa M. (2003). Ovarian blood flow responses to electro-acupuncture stimulation at different frequencies and intensities in anaesthetized rats. *Autonomic Neuroscience*.

